# Fabry–Perot Interferometric Fiber-Optic Sensor for Rapid and Accurate Thrombus Detection

**DOI:** 10.3390/bios13080817

**Published:** 2023-08-15

**Authors:** Marjan Ghasemi, Jeongmin Oh, Sunghoon Jeong, Mingyu Lee, Saeed Bohlooli Darian, Kyunghwan Oh, Jun Ki Kim

**Affiliations:** 1 Department of Physics, Yonsei University, Seoul 03722, Republic of Korea; m.g480@yonsei.ac.kr (M.G.); jsh2020@yonsei.ac.kr (S.J.); leemg3898@yonsei.ac.kr (M.L.); 2 Department of Biomedical Engineering, University of Ulsan College of Medicine, Seoul 05505, Republic of Korea; mini1kr@naver.com (J.O.); saeed.b.darian@gmail.com (S.B.D.); 3 Biomedical Engineering Research Center, Asan Institute for Life Science, Asan Medical Center, Seoul 05505, Republic of Korea

**Keywords:** thrombus detection, fiber optic sensor, fabry perot interferometer, biological sensing

## Abstract

We present a fiber-optic sensor based on the principles of a Fabry–Perot interferometer (FPI), which promptly, sensitively, and precisely detects blood clot formation. This sensor has two types of sensor tips; the first was crafted by splicing a tapered fiber into a single-mode fiber (SMF), where fine-tuning was achieved by adjusting the tapered diameter and length. The second type is an ultra-compact blood FPI situated on the core of a single-mode fiber. The sensor performance was evaluated via clot-formation-indicating spectrum shifts induced by the varied quantities of a thrombin reagent introduced into the blood. The most remarkable spectral sensitivity of the micro-tip fiber type was approximately 7 nm/μL, with a power sensitivity of 4.1 dB/μL, obtained with a taper fiber diameter and length of 55 and 300 μm, respectively. For the SMF type, spectral sensitivity was observed to be 8.7 nm/μL, with an optical power sensitivity of 0.4 dB/μL. This pioneering fiber-optic thrombosis sensor has the potential for in situ applications, healthcare, medical monitoring, harsh environments, and chemical and biological sensing. The study underscores the scope of optical technology in thrombus detection, establishing a platform for future medical research and application.

## 1. Introduction

Thrombosis is a clinical condition characterized by the formation of blood clots within the vasculature, thereby hindering blood flow. This can not only lead to cyanosis, which manifests as a bluish discoloration owing to oxygen deprivation but can also induce necrosis (the death of tissue due to lack of nourishment). Moreover, an extended state of thrombosis may trigger a prolapse of blood vessels, which further promotes thrombosis and exacerbates the general interruption of blood supply to the tissues. Importantly, thrombosis can cause serious life-threatening events, such as stroke and myocardial infarction. Particularly, venomous thrombosis manifests in approximately 1–3 in 1000 individuals annually. This translates to an approximate total of 300,000–600,000 cases yearly in the US, emphasizing the significant health burden it represents [1,2].

Thrombus formation is a complex process that involves several coagulation factors, including thrombin, tissue factor (TF), and fibrinogen [3,4,5]. Among these, thrombin maintains a fine balance between bleeding and clotting in the hemostatic system of the body [6]. Primarily, thrombin functions to promote hemostasis, thus averting excessive bleeding post-vascular injury. However, dysregulated thrombin formation or activity can disrupt this equilibrium, encouraging clot formation and potentially inducing thrombosis [7].

Understanding and managing thrombus formation and its subsequent ramifications on health are paramount. Both arterial and venous thrombi have serious potential consequences, including heart attacks [8], strokes [9], deep vein thrombosis (DVT) [10], and pulmonary embolism [11]. These diverse outcomes underline the remarkable impact of thrombosis on individual health and the extensive burden it places on the healthcare system.

Despite medical advancements, the prognosis of thrombosis remains challenging owing to the intricacies of its pathophysiology and inherent constraints in the present clinical diagnostic systems [12]. This highlights the importance of adopting comprehensive disease management strategies. It is crucial to not only address the immediate thrombotic incident but also to incorporate continuous monitoring of critical factors, such as thrombin, into the therapeutic regime. Practicing such a holistic approach yields a nuanced interpretation of a patient’s coagulation status and could potentially enhance prognostic accuracy. Furthermore, it could help develop more specific and efficacious therapeutic interventions for thrombosis.

Various techniques have been employed to monitor coagulation, including acoustic methods [13,14], Raman scattering spectroscopy [14,15], colorimetric analysis [16], deep learning algorithms [17], and near-infrared fluorescence imaging [18]. However, fiber-optic sensors are a particularly effective approach as they allow quick and real-time monitoring of thrombin [19,20,21]. Fiber sensors have practical applications in medicine, including cancer therapy [22], magnetic resonance imaging (MRI) [23,24], catheter-tip sensors [25], and potential health risks associated with exposure to microwaves [26].

Several extant fiber-based optical sensors can measure temperature, pressure, and strain only [21,27,28]. Hence, there is an opportunity to create a fiber sensor that utilizes a coagulation factor modality for clinical uses. Various optical structures have been developed for sensor fabrication, including fiber Bragg gratings [29], Mach–Zehnder interferometers [30], Michelson interferometers [31], Fabry–Perot interferometers [21,32], and other designs. Among them, Fabry–Perot interferometers (FPIs) receive considerable attention owing to their outstanding performance, compact size, simple configuration, and high sensitivity [33,34]. In addition, FPIs are unsusceptible to electromagnetic interference [35], making them particularly appealing for various applications. An FPI optical fiber sensor uses light wave interferences to detect alterations in the physical parameters [36]. The light passes through the fiber and travels back and forth between the reflective surfaces. These reflections form constructive and destructive patterns of light waves, which are analyzed to identify the changes in the monitored physical parameters.

According to this research, the creation of a Fabry–Perot sensor using a short segment of microfiber (MF) and single-mode fiber (SMF) is proposed [37,38] to detect blood coagulability by thrombin within a specific range. MF is generated by tapering an SMF [37,39] to a diameter of 55 µm and a length of 330 µm. By splicing a short MF section to a cleaved end of an SMF, an MF Fabry–Perot cavity is formed. Another type is the ultra-compact blood FPI on the core of a single-mode fiber with a diameter of 8 µm [21]. The results suggest that the sensor detects coagulation [40] by observing the shift in the interference fringe as the thrombin volume increases [41]. The sensitivities of the micro-tip and SMF tip sensors are reported to be 7 and 8.7 nm/μL, respectively. This approach presents a unique opportunity to further explore the mechanisms of thrombosis.

**Figure 1:**
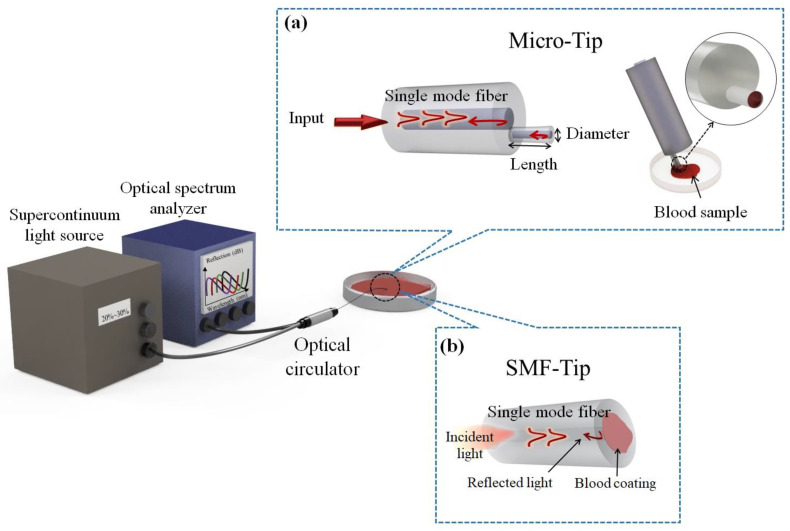
Schematic configuration of experimental set-up based on Fabry–Perot interferometer for blood monitoring: using (**a**) micro-tip (single-mode fiber (SMF)-taper base) and (**b**) SMF tip configuration.

The configuration of an FPI sensor is shown in Figure 1. The process involves immersing the prepared fiber into a blood sample. The hydrophilic properties of the silica surface allow the formation of a droplet of liquid on the end face of the fiber. Proper illumination must be maintained during the dipping process to ensure that the blood effectively coats the fiber core.

## 2. Materials and Methods

### 2.1. Sample Preparation

Thrombin activation triggers blood clot formation. Prothrombin is activated by calcium ions and converts into thrombin. Thrombin activates fibrinogen to make fibrin, which traps red blood cells and debris within its net-like structure and forms a blood clot.

To simulate thrombus formation in the vein, artificial in vitro thrombus formation was induced in the citrated sheep blood sample (Kisan Bio Co., Seoul, Republic of Korea). To induce thrombus in citrated blood, we used bovine thrombin reagent (Goan Medical Co., Seoul, Republic of Korea), and CaCl_2_. Citrated blood needs calcium ions to coagulate because the citrate ion forms calcium citrate. CaCl_2_ was added to supply calcium ions. The lyophilized thrombin (1000 mg) was reconstituted using approximately 34 mL of distilled water. To ensure its stability and long-term storage, the reconstituted thrombin was aliquoted and preserved at −80 °C until further use [41].

**Figure 2:**
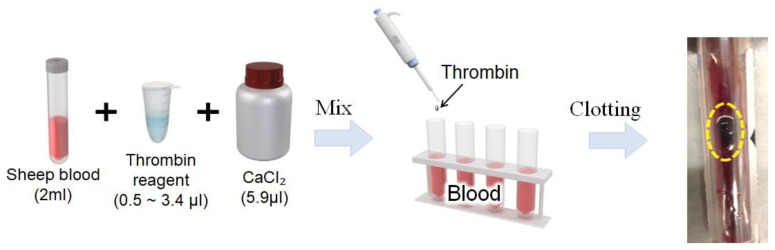
Efficacy of bovine thrombin in blood clotting: a volume-dependent assessment with citrated sheep blood.

The effectiveness of the thrombin reagent was assessed using citrated sheep blood (Kisan Bio Co.). As shown in Figure 2, varied volumes of thrombin, ranging from 0.5 μL to 3.4 μL, were mixed with CaCl_2_ (final concentration = 5.9 μL) and 2 mL sheep blood within a 30 min window to create eight discrete samples. Rapid clot formation was observed within minutes post-mixing, underlining the high efficacy and efficiency of the thrombin reagent-enabled coagulation process.

### 2.2. Sensor Fabrication and Operation Principle

The fiber-tip-FPI structure is an incredibly powerful and highly sensitive device that has revolutionized the field of refractive index measurement. Its unique design, consisting of an SMF and MF cavity [21,37], facilitates accurate and precise detection and measurement of changes in the refractive index. By generating interference between the light waves traveling through the SMF and those traveling through the MF cavity, the tip-FPI creates a pattern of bright and dark fringes that shifts with the changes in the refractive index or cavity length.

Sensitivity to the smallest changes in the refractive index or cavity length makes the tip-FPI an invaluable tool for numerous applications, from environmental monitoring to medical diagnostics. Its ability to accurately detect and measure changes in the refractive index provides critical insights into the behavior of various materials and substances, allowing researchers and practitioners to make informed decisions about their best utilization.

The interference phenomenon produced by the crossing of two light beams at the junction of SMF and MF can be described using the two-beam optical interference Equation (1) [42].

(1) $$I=I_{1}+I_{2}+2\surd I_{1}I_{2}cos(\delta )$$

where *I* is the intensity of the interference signal, and *I_1_* and *I_2_* are the reflection intensities forming the two interfaces, respectively. This equation considers the reflection intensities at the two interfaces and the phase difference ∆*δ* between the two reflection beams. The points of minimum interference occur when the phase difference is equal to an odd multiple of *π*, particularly (2*m* + 1) *π*, where *m* represents an integer.

The central wavelength of the *m*th order interference dip is given by Equation (2):

(2) $$\lambda_{m}=4\pi n_{MF}\frac{L}{\left(\left(2m+1\right)\pi -\varphi_{0}\right)}$$

where *n_MF_* is the refractive index of the *MF*, *L* is the length of the *MF*, and $\varphi_{0}$ is the initial phase of the interference. This equation is especially useful as it allows the calculation of the central wavelength of the interference dip for any given order of interference. This information is crucial for determining any shifts in the interference spectrum that may occur due to variations in the cavity’s length or refractive index.

Free spectral range (*FSR*) is the spectral fringe spacing between adjacent interference notches and is given by Equation (3) [43]:

(3) $$FSR=\frac{{\lambda_{m}}^{2}}{2n_{MF}L}$$

*FSR* is a fundamental parameter of the tip-FPI structure because it describes the spectral range over which interference fringes are observed; therefore, knowing that *FSR* is necessary to interpret the shifts in the interference spectrum properly.

The contrast of the interference fringe is determined by the intensity of the two light beams and can be controlled by adjusting the relative cross-sectional position. This ability to control the interference fringe contrast is a valuable feature of the tip-FPI structure, as it imparts greater sensitivity in measuring changes in the refractive index [21,36,44].

**Figure 3:**
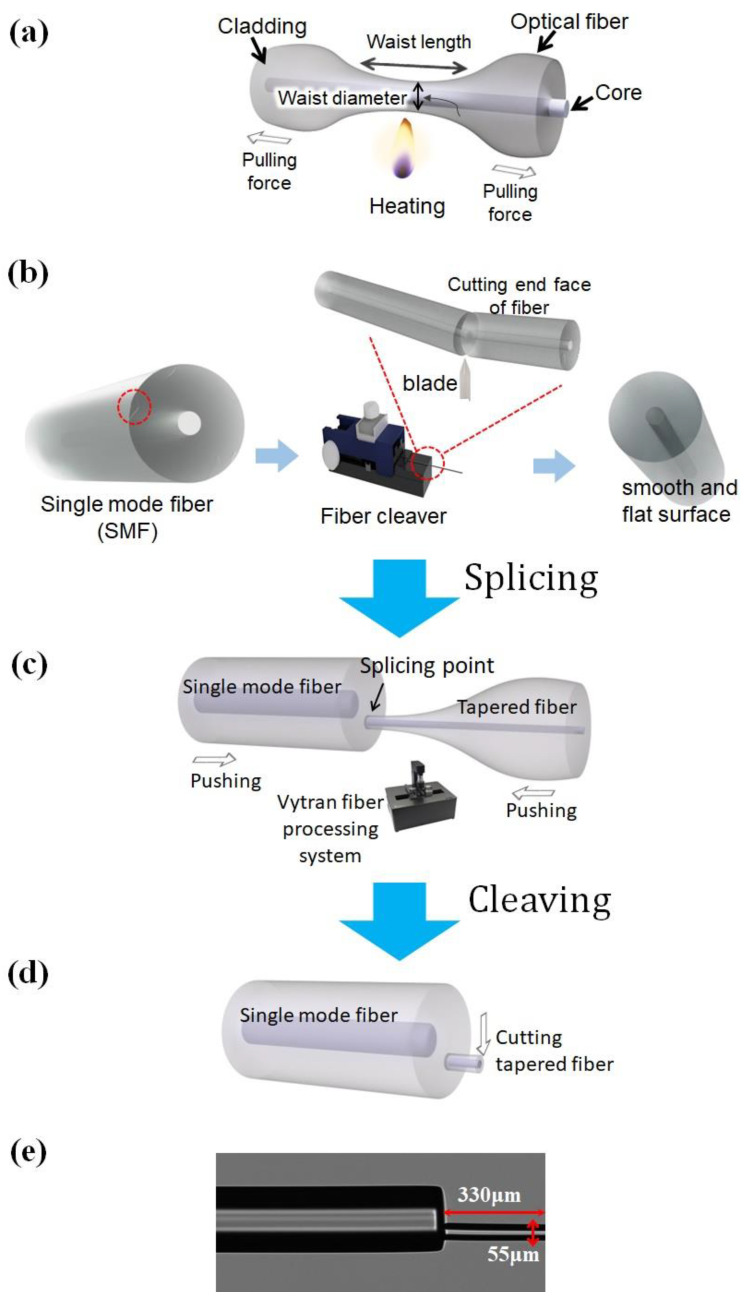
Fabrication process of MF tip-FPI. (**a**) Tapering single-mode fiber (SMF). (**b**) Process of fiber cleaving. (**c**) Splicing tapered fiber to SMF. (**d**) Cleaving the end side of tapered fiber to obtain a structure based on SMF-tapered fiber. (**e**) Microscopic view of micro-tip-FPI.

Fabrication of tip-FPI is a critical and meticulous procedure that involves several essential steps (Figure 3). Each step is executed carefully to ensure the highest quality and reliability of the fabricated tip-FPI.

As shown in Figure 3a, the process begins with the tapering of a standard SMF using a Vytron machine [45,46]. It heats a section of the fiber and pulls it apart using a precise tension control system. This process gradually reduces the diameter of the fiber over a specific length, resulting in a tapered fiber. The MF diameter is controlled by adjusting the elongation speed and travel distance, and the tapered fiber is then cleaved into two segments at its waist position, creating a smoothly cleaved end (Figure 3b).

As shown in Figure 3c, the pigtail of the cleaved tapered fiber is spliced onto a cleaved SMF at an optimized relative cross-sectional position using a commercial Vytron GPX-3000 glass-processing and fusion-splicing machine. The splicing position has a greater influence on the interference fringe contrast than on the sensing properties. Therefore, an arc power and a duration time are set to fabricate an optimized diameter for 55 µm MF tip-FPI. The tapered fiber is offset by approximately 30 μm from the center of the SMF core (Figure 3d) [37]. Figure 3e shows the microscopic view of the MF-tip.

The MF is cleaved at the designed length (330 µm) to make the tip-FPI. Notably, a large diameter may cause heavy losses due to the reduction in the energy density of the reflected light [47,48], whereas a small diameter would significantly increase the fabrication complexity in both cleaving and splicing.

Tapered fiber has a smaller mode field diameter (MFD) than standard fibers, which results in greater light intensity at the fiber tip. Additionally, the gradual reduction in diameter ensures a smoother transition between different regions of the fiber, reduces scattering, and improves overall fiber performance. This makes them ideal for applications requiring high optical power, such as fiber lasers and nonlinear optics.

The FP cavity of the SMF tip is generated by placing a blood layer over the optical fiber’s end face. As shown in Figure 1a, it creates two distinct reflective interfaces. The first is established at the boundary between the optical fiber core and the blood, where a change in refractive index causes a portion of the light to reflect back into the fiber while the remainder continues into the blood. The second interface forms at the boundary between the blood and ambient air, where a similar refractive index shift leads to another portion of the light being reflected. This light then travels back through the blood, with a portion of it reflecting again at the initial interface and subsequently propagating back through the fiber.

The innovative, real-time thrombin detection system highlighted in this study uses the distinct capabilities of an advanced Fabry–Perot interferometer (FPI) to measure variances in light reflection. It can detect thrombin levels ranging between 0.5 and 3.4 μL, a domain of significant relevance in several clinical settings, thus underscoring the potential broad-spectrum application of this pioneering technology.

The system design includes a supercontinuum light source (NKT Photonics, EXR-15, Birkerød, Denmark), a fiber optic circulator (Thorlabs, SMF, FC/APC, Newton, NJ, USA), and an optical spectrum analyzer (Agilent, 86140B) to accurately gauge the reflective spectral response of the tip-based FPI. As shown in Figure 1, the configuration emphasizes fluid integration of these components to create a robust and reliable sensing platform.

## 3. Result

### Comparative Analysis: SMF Tip vs. Micro-Tip

**Figure 4:**
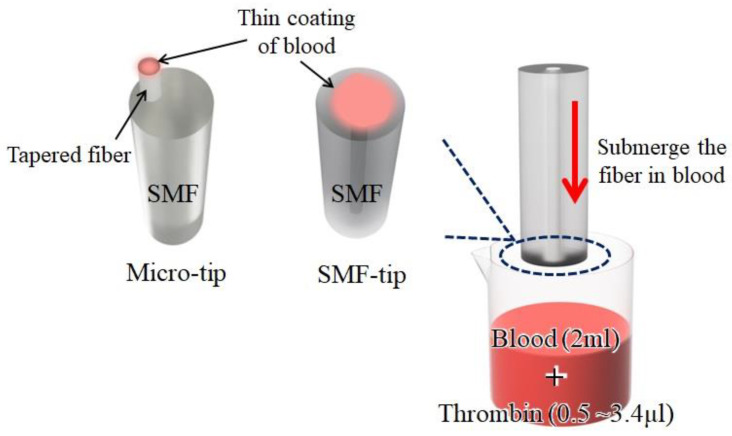
Configurations of creating a blood layer on a fiber facet.

One key feature of the system is its ability to recognize the minute changes in the reflected light from the sensor head when exposed to varying thrombin levels. As shown in Figure 4, when the device is immersed in a blood sample, the signal intensity diminishes due to the attenuated refractive index contrast, which, in turn, results in lower Fresnel reflections from the cavity end-faces [49,50]. Simultaneously, the reduced spectral distance between adjacent valleys in the interference spectra suggests an increase in the refractive index of the medium inside the cavity, further evidencing the presence of thrombin in the blood.

Moreover, the immersion of a fiber sensor in a blood sample can alter the effective length of the Fabry–Perot cavity. Minor changes in the blood layer’s thickness can affect the refractive index and the cavity’s length, which in turn, influences the interference pattern. This is in line with the Fabry–Perot interferometer principle, where the interference type depends on the distance between two reflective surfaces.

**Figure 5:**
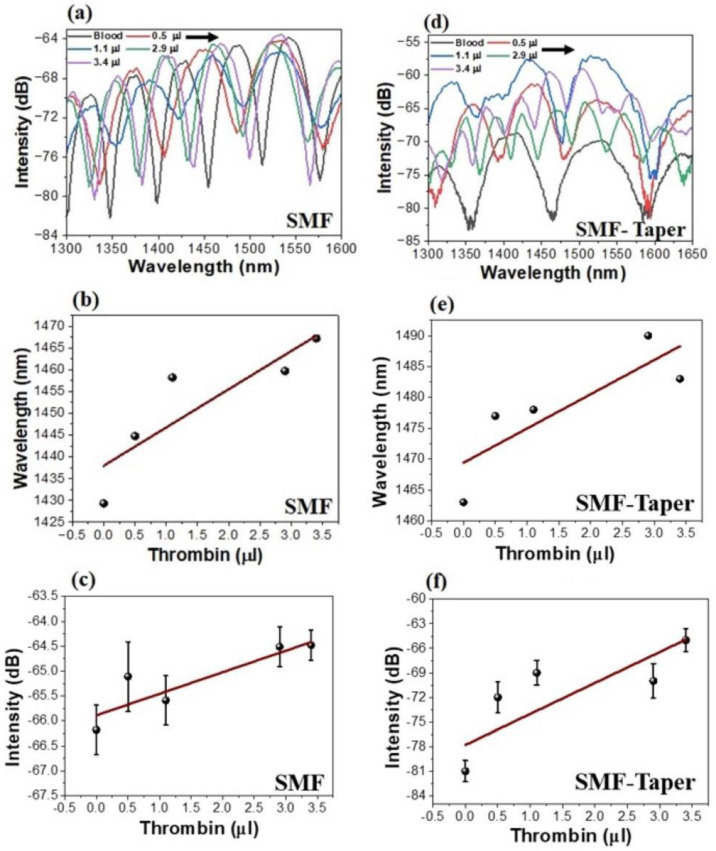
Thrombin-dependent spectra of the fabricated sensor. (**a**) SMF tip and (**d**) micro−tip (SMF −taper) from blood sample with a thrombin range of 0.5−3.4 μL. (**b**) Corresponding spectral shifts and (**c**) power changes for SMF tip. (**e**) Corresponding spectral shifts and (**f**) power changes for micro-tip.

We observed that the spectral sensitivity of our SMF tip-based thrombin-FPI sensor was slightly higher (8.7 nm/μL) compared to that of the micro-tip configuration (7 nm/μL). The results are shown in Figure 5a–f. This outcome may initially seem counterintuitive, considering that a tapered fiber should theoretically increase evanescent field interaction and, thus, enhance sensitivity. However, the obtained results opened interesting considerations.

In this study, the SMF tip demonstrated a more robust response to spectral shifts, despite its ostensibly low evanescent field. One potential explanation could be the MFD of the fibers. While tapering enhances the evanescent field, it also reduces the fiber MFD, which decreases the mode volume interacting with the biological sample. In contrast, SMF, with a larger MFD, maintains a larger mode volume for interaction, which may contribute to a stronger response to refractive index changes induced by thrombin addition [51,52].

Furthermore, the geometry of the SMF may have a more stable configuration, with less susceptibility to external perturbations, which can maintain reliable sensor performance. The simpler structure of the SMF sensor may also reduce potential variability that may be introduced during the fabrication process, resulting in more consistent performance.

**Figure 6:**
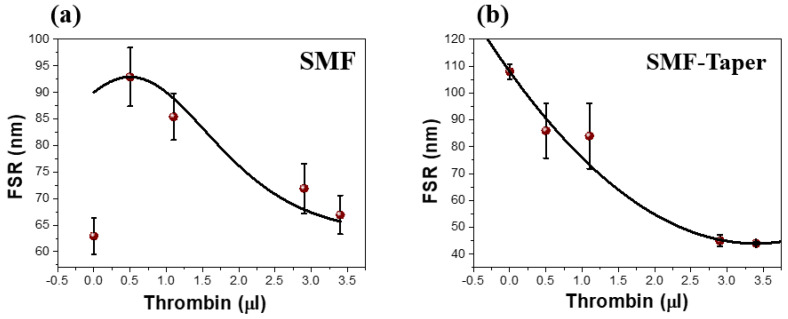
Simulation of FSR for (**a**) SMF-FPI sensor and (**b**) SMF−taper FPI sensor. Red circles refer to the experiment, and the black solid curve is the fitting curve.

Notably, our sensors perform optimally within the thrombin volume range of 0–3.4 μL. In Figure 6a,b, the free spectral range (FSR) of the FPI diminishes as the thrombin volume increases, leading to domination of the spectral shift on the narrow FSR [21,53,54]. This observation underlines the importance of careful management of the balance in the system to ensure precise measurement.

## 4. Discussion

Our investigation validates that the FPI-based sensor system described in this study effectively and accurately identifies the changes in the thrombin levels within the tested range, offering promising clinical applications. Early detection and accurate measurement of thrombin can play a vital role in diverse medical diagnoses and treatment protocols, ranging from managing coagulation disorders to optimizing surgical outcomes. Therefore, a broader application of this technology could significantly improve patient care and prognosis.

Our sensor development work has challenged traditional approaches and yielded remarkable results that have considerable potential in the field of bio-sensing, particularly in monitoring changes in thrombin volume. In the design of our FPI sensor, we used a standard SMF with core and cladding diameters of 8 and 125 μm, respectively. This was connected to a prepared taper with a total diameter of 55 μm and a length of 330 μm. The connection was offset by approximately 30 μm, a significantly larger value than the offset of 4 μm used in a previous study conducted by another group [37].

One might reasonably expect that the larger offset would lead to a smaller overlap between the mode fields of the SMF and taper, thereby reducing the interaction of the light with the biological sample and resulting in a lower spectral sensitivity. However, our sensor exhibited higher spectral sensitivity, despite this larger offset.

Several factors could potentially explain this result. First, the larger offset may result in a broader evanescent field, which increases the interaction of light with the sample despite the decreased core overlap [51,55,56]. Consequently, this could lead to a stronger response to the changes in the refractive index of the blood caused by the addition of thrombin. Second, the SMF tip used in our design may provide a larger mode volume for interaction with the sample, as previously discussed. Although the lower theoretical evanescent field compared to that of a tapered fiber, the larger mode volume may result in a stronger response to refractive index changes, leading to higher spectral sensitivity [57].

Notably, the previous study was focused on temperature sensing [37], whereas our work is focused on sensing changes in thrombin volume. The optimal sensor design may differ for these two applications due to the differences in the nature of the physical changes being detected. In our case, the addition of thrombin leads to changes in the blood refractive index due to coagulation and fibrin formation. These changes may be more effectively detected using an SMF tip sensor with a larger offset, as we have demonstrated in this study.

The performance of a FPI sensor in a biological context depends on a delicate balance between physical, chemical, and optical phenomena [57,58]. The introduction of thrombin into a blood sample triggers coagulation, which alters the physical properties of the blood, such as viscosity, and leads to fibrin formation. These changes may slightly modify the refractive index of the blood [59,60], a critical parameter affecting the optical path length within the FPI and, consequently, influencing the interference pattern.

## 5. Conclusions

In conclusion, the development of a highly effective and compact FPI sensor by splicing MF to the end of an SMF and an ultra-compact blood FPI on the core of a single-mode type represents a significant breakthrough in sensor technology. The miniaturized sensor not only displays comparable linearity but can also be easily manufactured, making it a perfect alternative for detecting specific areas.

Furthermore, the sensing ability of the sensor remains excellent at increased thrombin volumes, and its spectral sensitivity of 7 nm/μL for micro-tip and 8.7 nm/μL for SMF tip in the thrombin range of 0.5–3.4 μL is noteworthy. These experimental results validate the theoretical analysis of the reflection mode of the tip-FPI, underscoring the potential of the proposed miniaturized sensor in various fields.

The remarkable characteristics and diverse applications of the compact FPI sensor make it a valuable tool for multiple applications, ranging from in situ healthcare monitoring to chemical and biological sensing. The current study elucidates the immense promise held by the convergence of optical technology and medical science, ultimately paving the way for more comprehensive and transformative medical diagnostics and monitoring strategies in the future.

## Data Availability

All data in this study are included in the article itself.
